# Mandrake: visualizing microbial population structure by embedding millions of genomes into a low-dimensional representation

**DOI:** 10.1098/rstb.2021.0237

**Published:** 2022-10-10

**Authors:** John A. Lees, Gerry Tonkin-Hill, Zhirong Yang, Jukka Corander

**Affiliations:** ^1^ MRC Centre for Global Infectious Disease Analysis, School of Public Health, Imperial College London, London W2 1PG, UK; ^2^ European Molecular Biology Laboratory, European Bioinformatics Institute EMBL-EBI, Hinxton CB10 1SD, UK; ^3^ Department of Biostatistics, University of Oslo, 0317 Oslo, Norway; ^4^ Department of Computer Science, Norwegian University of Science and Technology, 7491 Trondheim, Norway; ^5^ Aalto University, 02150 Espoo, Finland; ^6^ Parasites and Microbes, Wellcome Sanger Institute, Cambridge CB10 1SA, UK; ^7^ Helsinki Institute for Information Technology HIIT, Department of Mathematics and Statistics, University of Helsinki, 00100 Helsinki, Finland

**Keywords:** pathogens, population structure, visualization, genomics, dimensional reduction

## Abstract

In less than a decade, population genomics of microbes has progressed from the effort of sequencing dozens of strains to thousands, or even tens of thousands of strains in a single study. There are now hundreds of thousands of genomes available even for a single bacterial species, and the number of genomes is expected to continue to increase at an accelerated pace given the advances in sequencing technology and widespread genomic surveillance initiatives. This explosion of data calls for innovative methods to enable rapid exploration of the structure of a population based on different data modalities, such as multiple sequence alignments, assemblies and estimates of gene content across different genomes. Here, we present Mandrake, an efficient implementation of a dimensional reduction method tailored for the needs of large-scale population genomics. Mandrake is capable of visualizing population structure from millions of whole genomes, and we illustrate its usefulness with several datasets representing major pathogens. Our method is freely available both as an analysis pipeline (https://github.com/johnlees/mandrake) and as a browser-based interactive application (https://gtonkinhill.github.io/mandrake-web/).

This article is part of a discussion meeting issue ‘Genomic population structures of microbial pathogens’.

## 1. Introduction

Advances in DNA sequencing technology have recently made whole-genome sequencing both affordable and scalable enough for routine use in pathogen surveillance by research organizations and public health agencies around the world [1,2]. A striking example of this is genomic surveillance of the SARS-CoV-2 virus for which over one million genome sequences became available in just 15 months after its initial discovery [3]. To shed light on population genomic data at this scale calls for new tools that can be used for rapid exploration of the structure among the samples, with particular emphasis on detecting clusters of similar sequences [4,5]. In this paper, we explore and extend a class of methods that aims to reduce the dimensionality of such data to only two dimensions, in a manner that supports ready visualization and identification of clusters.

Finding clusters of genetically similar samples has a number of uses, for example: identifying plausible transmission pairs and determining patterns of global dissemination, and as a proxy for traits such as virulence, host range and antimicrobial resistance [6,7]. Ideally clusters will have direct biological relevance, either through one of these uses or by representing a meaningful evolutionary unit (often referred to as a strain or a lineage, though neither of these terms have a consistently used formal definition) [8].

While we do not aim to comprehensively address the clustering problem here, we do consider some of these methods as useful comparisons, so review these briefly. Most methods for clustering genomes fall into one of four categories: likelihood-based, phylogeny-based, ‘gene-by-gene’-based and distance-based. Likelihood-based methods determine clusters by fitting a multinomial model to allele frequencies based on a sequence alignment [9]. Phylogeny-based methods usually assign selected clades in a phylogenetic tree as clusters [5]. Gene-by-gene approaches assign an integer identifier to each unique DNA sequence of a gene that has been observed and an overall identifier for each unique set of gene identifiers a genome possesses [10]. These identifiers can be used as clusters directly, but often more useful clusters can be achieved by linking samples that share some proportion of their gene labels [11,12]. Distance-based models use pairwise genetic distances between all samples as input [13] and sometimes also apply similar approaches of linking similar samples [14].

One challenge with applying any of these methods is that many species do not have good quality schemes to label input genomes, or suffer from poor-quality or missing metadata. This makes unsupervised methods of particular interest when exploring data [15]. A further challenge arises from the size of the data we wish to process. On top of the fact that we wish to process a large number of individual genomes, genomic datasets typically have a very large number of features; for example when using SNPs or *k*-mers to represent sequence variation, each sample may typically have 10^6^–10^8^ such markers. These markers are frequently used to calculate genetic distances between samples, the number of which grows as the number of samples squared, such that one million samples will have of the order of 10^11^ distances between them. Such high dimensionality of population genomic data is beyond the capability of most analysis methods available today, rendering it difficult to gain insight into the data structure in a fast and robust manner.

One approach to understanding structure in this size of data is to embed the samples into a space that can easily be visualized, which can be both generalizable and fast. An embedding seeks to find a lower-dimensional representation of data where the distances in the lower-dimensional space *y* (output) are an accurate representation of distances in the higher-dimensional space *×* (input). Intuitively, genetically similar samples should be close together in the embedding space, and genetically distant samples should be further apart in the embedding space. Embedding spaces may be linear combinations of the input dimensions as in principal component analysis and multi-dimensional scaling, but here we focus on nonlinear methods, which can infer potentially complex manifolds relating input to output spaces in an unsupervised data-driven manner. This means, unlike in linear methods, the transform in one part of the input space may be quite different from another part of the space.

Probably the most well-known method used to find lower dimensional embeddings of data is t-distributed stochastic neighbour embedding (t-SNE) [16,17]. t-SNE and related methods have been used extensively to represent and visualize data from numerous fields of research, and they have recently been considered for analysing population structure in both human and pathogen populations, as well as data from single-cell genomics [18–21]. As these are unsupervised methods, they do not use sample labels to find the embedding. Due to the choice of the output probability distribution, distances between local samples are preserved, whereas global distances are less well preserved. Consequently, t-SNE is often used to identify clusters in high-dimensional data that may correspond to units of population structure such as species, strains or lineages. Alternatively they may map onto sample labels, such as their geographical origin or cell type.

However, t-SNE is not optimizing the embedding to find clusters. So, when clusters do emerge, they are an indirect consequence of preserving local structure in the data. The recently developed method of stochastic cluster embedding (SCE) [22] generalizes t-SNE to include an additional scaling parameter, which was selected on the basis of a user survey where participants were asked to rate how clustered data appeared to be. The authors showed that this scale factor can be chosen to exactly replicate t-SNE, or alternatively can be tuned to effectively increase the ‘repulsion’ between points, targeting distinct clusters forming in the output embedding, which are easier to visualize and interpret.

In this paper, we extend the SCE method to use a variety of genomic data modalities as input, improve its performance on large datasets and add a range of output visualizations. Our method allows users to rapidly gain insights into structure present in very large genome datasets, which we show corresponds well with model-based genetic clustering algorithms. We implemented our method as a piece of open-source software called mandrake (https://github.com/johnlees/mandrake) and as a static web application (https://gtonkinhill.github.io/mandrake-web).

## 2. Methods

### (a) Calculating between-sample distances from genome data

As input, Mandrake takes one of three types of data: a multiple sequence alignment, a set of *k*-mer sketches (can be created from assembled or sequence read data) or a binary presence–absence matrix (which is typically used to represent genes, but can be used to represent other genetic elements). These are all treated in fundamentally the same way, as feature matrices, with *N* samples (genomes) along rows and *M* features (SNPs, *k*-mers or genes) along columns. Although typically genomic datasets have been 'wide', with many more features than samples, the scale of data means this is no longer the case and we are now able to analyse the case with more samples than genomic features.

To calculate input distances **X** from the feature matrix **A**, we can compute **X** = *M* − **AA**^T^, which counts the number of shared features between every pair of samples (the similarity), **AA**^T^, and converts this to a distance by subtracting from the maximum shared features *M*. This is a symmetric matrix with zeros on the diagonal. We note that more sophisticated genetic distance calculations are possible by accounting for base frequencies and varying transition rates between classes, but we do not consider such distances here.

A difficulty is that both the number of calculations needed to find **X** and the amount of memory to store **X** grow as *N*^2^. Here we use methods that are fast enough to scale to *N*^2^ for at least one million samples, but such a matrix would still require at least 2Tb of memory (or disc space). To avoid this major resource issue, we cut the size of **X** down using one of two methods. The first is to set a distance threshold above which entries from **X** are discarded. The second, which we use for all analyses here, is to retain just the *k* nearest neighbours for each sample (excluding self distances, and including any ties). This means **X** grows linearly in size with *Nk* in a predictable way, making memory allocations efficient. As the perplexity sets the expected number of neighbours, choosing a *k* above the desired perplexity will typically give good results. In practice, we store **X** as a sparse matrix in coordinate (triplet) format, with three ordered lists of row-index *i*, column-index *j* and *x_ij_* for each retained distance. We save these to disc so they can be re-used by other programs, or by Mandrake to re-run the embedding without recomputing distances.

When **A** is a multiple sequence alignment, we code each row using the four DNA bases, each in its own dynamic bitset with the same length as the alignment, storing 1 if the base is present in that sample at that position, and 0 otherwise. Elements $x_{ij}=M-\Sigma a_{im}a_{\;jm}$ are then computed by ANDing each of the four bitsets and counting the total number of bits that are on (popcount). The use of bitsets ensures efficient packing into 64-bit words, which makes the Boolean AND operation and subsequent popcount very fast to complete across all *M* sites. If **A** is a gene presence/absence matrix, the procedure is similar, but only a single bitset is needed for each gene.

For sequence assemblies or sequence reads that are unaligned, we count the number of shared *k*-mers between samples. Reads can be 'cleaned' by first removing low-frequency *k*-mers, which typically are a consequence of sequencing error. Rather than using all *k*-mers, of which there are a prohibitively large number [23], we use a ‘sketching’ approach pioneered by the popular Mash software, which instead uses a hash function (a hash function here transforms a *k*-mer sequence to a 64-bit integer) to uniformly subsample a fixed-size subset of the total *k*-mers [24]. The proportion of shared *k*-mers (the Jaccard distance) can be computed by the size of the intersection of the retained hashes. We use two further modifications to this process. First, we use the method of BinDash [25] to bin hashes and calculate distances between them (which turns out to be very similar to the dynamic bitset approach, but using bits of the calculated hash instead of DNA bases). Second, we optionally enable the approach of PopPUNK, which calculates the Jaccard distance at multiple *k*-mer lengths and regresses their depletion at longer lengths to calculate core and accessory distances within a species [14]. In practice, we use PopPUNK's sketching and distance library pp-sketchlib (https://github.com/johnlees/pp-sketchlib), which optimizes sketching and distance calculation from assembly or read data and has an API that can be directly called from python.

The computation of each row of **A** and reduction to the *k*-nearest neighbours is embarrassingly parallel across up to *N* processes. We use OpenMP to achieve CPU parallelism. pp-sketchlib can also make use of CUDA compatible graphics processing units (GPUs) for further parallelism.

### (b) Stochastic cluster embedding

We start with a brief overview of t-SNE, which SCE then seeks to generalize. Rather than minimizing a scalar distance between the input and output data, t-SNE minimizes the Kullback–Leibler divergence between two probability distributions defined by the input and output data. In this section, we refer to samples (genomes) as rows of **X**, which can be indexed by an integer between 1 and *N*: *i*, *j*, *k* or *l*. The input distribution is a conditional probability distribution 0 ≤ *p*_*j|i*_ ≤ 1 between every pair of samples (genomes) *i* and *j,* which is defined for a given pair *i* ≠ *j* by

$$p_{\;j|i}=\frac{\exp(-||x_{i}-x_{j}|{|}^{2}/2\sigma_{i}^{2})}{\sum_{k\neq i}\exp(-||x_{i}-x_{k}|{|}^{2}/2\sigma_{i}^{2})},$$

To calculate this probability, it is necessary to set a value for the kernel variance $\sigma_{i}$ for each sample *i*. Intuitively, *p*_*j*|*i*_ then equals the probability that *x_i_* would pick *x_j_* as its neighbour when sampling from a normal probability distribution centred at *x_i_* with variance $\sigma_{i}$. Typically the kernel variance is not set directly for each sample, but instead an overall perplexity *K* is set by the user, which is defined for a discrete probability distribution *p* by raising two to the power of its entropy (in bits). Generally, a perplexity of *K* in a distribution *p* over *N* neighbours means *p* provides the same surprise as if we were to choose among *K* equiprobable neighbours, which can also be interpreted as the expected number of neighbours for each sample. Lower values of perplexity favour more local structure, whereas higher values assign greater weight to the global structure. Given a desired perplexity level *K* for each sample, kernel variances for each sample $\sigma_{i}$ can be estimated using an optimization method that uses interval bisection to produce the desired perplexity for each sample [26]. Together, these steps convert distances in **X** to conditional probabilities **P**, which are sometimes referred to the entropic affinities, and overall this step is referred to as entropic affinity preprocessing. In Mandrake, we used the Cython implementation in scikit-learn, adding CPU parallelism over samples with OpenMP [27].

In the output space, t-SNE defines the probabilities *q*_*ij*_ using a Student *t*-distribution with one degree of freedom (a Cauchy distribution):

$$q_{ij}=\frac{{\left(1+||y_{i}-y_{j}|{|}^{2}\right)}^{-1}}{\sum_{k}\sum_{l\neq k}{\left(1+||y_{k}-y_{l}|{|}^{2}\right)}^{-1}}.$$

The use of a heavy-tailed distribution rather than a normal distribution allows points to be further apart without affecting the divergence too much and is also faster to compute.

A popular measure of discrepancy between two probability distributions *P*(*x*) and *Q*(*x*) is given by the Kullback–Leibler divergence, which in this setting is defined as a sum over values *P*(*i*, *j*) and *Q*(*i*, *j*) from all pairs of samples, as indexed by *i*, *j*:

$$\mathrm{KL}(P||Q)=\sum_{i\neq j}\;p_{ij}\log\frac{\;p_{ij}}{q_{ij}}.$$

The t-SNE algorithm minimizes this divergence iteratively, thus giving an embedding *y* with a probability distribution for between-sample distances that is as similar as possible to the probability distribution for between-sample distances in the higher-dimensional data *x*.

We now give a brief overview of the mechanism behind SCE, but note that full details are covered in the original publication [22]. We also based our implementation on the reference implementation available at https://github.com/rozyangno/sce, and note the main changes here. The main difference between the SCE algorithm and the t-SNE algorithm described above stems from the addition of a scaling factor *s,* which appears in the denominator with *q_ij_*. This allows the objective function to be minimized, *D* (the modified Kullback–Leibler divergence):

$$D(P||sq)=\sum_{i\neq j}\left[\;p_{ij}\log\frac{\;p_{ij}}{sq_{ij}}-p_{ij}+sq_{ij}\right].$$

to be written in terms of an attraction *J*_attraction_, repulsion *J*_repulsion_ and constant *C* with respect to *q|s*:

$$\begin{array}{rl} D(P||sq)=\; & J_{\mathrm{attraction}}+J_{\mathrm{repulsion}}+C\\ J_{\mathrm{attraction}}=\; & \sum_{i\neq j}\;p_{ij}\log q_{ij}\\ J_{\mathrm{repulsion}}=\; & s\sum_{i\neq j}q_{ij}\\ C= & -\log s-1+\sum_{i\neq j}\;p_{ij}\log p_{ij} \end{array}\}$$

In the previous SCE study, it was noted that when *s* is the normalizing factor for *q* this optimization is exactly equivalent to t-SNE. However, when *s* is increased this adds extra repulsion, typically forming tighter and visually clearer clusters, which is similar to the 'early exaggeration' step in many t-SNE implementations. SCE picks a larger value for s (see paper for formulae) to form clearer clusters. The authors confirmed that their chosen value was a good choice through a user study, where participants used a slider for s to select a value that best explained the clusters in four datasets from a variety of sources, and found that the mean value across users was closer to the SCE choice than the t-SNE choice.

The SCE method optimizes *D* using stochastic gradient descent (SGD), a popular method to fit neural networks [28]. Here, the output embedding **Y** is updated given the current *s*, then *s* is recomputed using the update **Y**. This is repeated for a specified number of iterations, chosen such that *D* reaches a stable minima. To stochastically update **Y** at each iteration, a pair of samples *i*, *j* are chosen at random in proportion to their conditional probabilities *p*_*j|i*_, and the gradient ▽ of their attraction term calculated (such that *C* can be ignored). Then, a second pair of samples *k*, *l* are chosen at random in proportion to the sample weights (which by default is equal for every sample) and the gradient of their repulsion term calculated. In SGD, a learning rate η is used to update **Y** by making a small step down the direction of the gradient $y_{t\;+1}\leftarrow y_{t}-\eta_{t}▽$ at iteration *t*. The learning rate decreases across the total *T* iterations *T* as $\eta_{t}=\eta_{0}\cdot (1-t/T)$. Larger steps are taken in early iterations, and smaller steps are taken in later iterations closer to convergence. **Y** is initialized by drawing $y_{0}∼U(0,10^{-4})$ along each dimension for each sample, repeating draws which lie outside of a circle with radius 10^−4^ centred at the origin.

While an additional drawback of t-SNE was that the iterative optimization is challenging to directly scale to larger datasets, SGD is simpler to parallelize. At each step updating **Y**, *w* workers can independently pick two pairs of points *i* and *j; k* and *l* to update. Ideally for CPU parallelizm, *w* will be chosen equal to the number of physical cores, and for GPU parallelism w will be chosen to be large (10^5^ or more) to maximize device occupancy. A potential issue arises if two workers try to update the same sample at the same time (bearing in mind the additional complication that these workers may not be in sync). This becomes more likely when the number of active workers is not much less than the number of samples. We address this in the CPU implementation by using atomic operations to preserve memory integrity, and when overwritten by another worker, retry with another pair. CUDA global memory is not directly affected by memory integrity issues from race conditions, but we still use an atomic operation to update **Y** rather than a simple overwrite. In each case, as long as memory integrity is preserved, the stochastic nature of the algorithm will correct for missteps in subsequent iterations, as long as they do not dominate. Additionally, while atomic operations are faster than locks, they become slower when multiple threads are attempting to operate on the same memory address, leading to a reduction in efficiency. We therefore output the proportion of workers found to be 'clashing' at each iteration, so users are aware they may wish to lower *w* when analysing smaller *N*.

We also note that we use the method of Walker [29] for drawing discrete random variables to precompute tables to draw edges from *P_j|i_* in constant time, reimplementing the GSL library implementation in C++ [30]. We also use the fast parallel random number generator from the dust package [31], which is based on the xoshiro128+ generator [32], and can be used to produce uncorrelated pseudorandom 32-bit integers in parallel on both CPUs and GPUs. This also removed all link time dependencies from the compiled code, which made compilation into WebAssembly straightforward (see below).

### (c) Visualizing embeddings

We automatically output the final embedding **Y** in four formats:

— A simple text file with *N* rows and two columns, for reuse by other programs or plotting software. A separate file listing sample names, and optionally clusters, is also created.
— An interactive HTML plot using the WebGL mode of plotly [33]. This can be viewed in a web browser, and scales up to millions of points. Embedding positions and labels appear on hover. For smaller datasets sample names also appear on hover, but this can be turned off (as resulting files can be extremely large on disc).
— A static image using matplotlib [34].
— A dot network file, which can be loaded for interactive viewing along with sample labels in Microreact [35].

To add colour to samples in the plot, the user can either provide labels, or labels can be generated by performing a spatial clustering on the embedding. For the latter, we use HDBSCAN, as this usually works well on well-separated clusters of unspecified shape. We centre and normalize the embedding to [−1, 1] in each direction, use a minimum cluster size of two and minimum distance between clusters of 0.02 [36]. HDBSCAN may label some points as ‘noise’, which are useful for potential singleton clusters, though care should be taken not to group noise points into a cluster.

Colours for classes are chosen by randomly sampling from RGB space. We tried selecting from HSL or HSLuv space, which are perceptually uniform colour spaces to the human eye, but found empirically that contrast between labels was poorer than from RGB colours.

We found that for many of the genomic datasets we ran Mandrake on, well-separated clusters were a common feature (for example separating species). In the embedding output, this leads to many points overlapping, and although clusters can clearly be identified, their size is obscured. To help remedy this, we included an additional (static) hexagon density plot which shows a heatmap of the number of samples in each region of the plot.

We also include code to create a video of the embedding process as the SGD algorithm runs, which is particularly useful for monitoring convergence. We take the current embedding and objective function at 400 points across the total number of iterations, create a static plot and use these as frames in the output animation (at 20 fps, so videos are 20 s in duration). In the CUDA code, the copy operation for the current embedding is launched asynchronously to the main SGD kernel run, so it has a negligible impact on run time. We optionally add sound by mixing decaying triangular wave oscillators at a frequency proportional to the maximum movement along each dimension between each frame. This sound is in stereo, with each channel corresponding to an SCE dimension.

Initially, our code sampled frames uniformly from the SGD iterations; however, this led to animations where at the start points moved too fast, and at the end too slow. This is due to the decreasing learning rate η. We decided instead to sample uniformly from the total amount of learning completed, so when more learning (and larger changes to the embedding) was being done more frames would be taken, and when less learning (and smaller changes to the embedding) was being done fewer frames would be taken. As we use a linearly decreasing learning rate, learning grows quadratically, so we sample proportional to its inverse (the square root).

### (d) Software implementations

Mandrake is written in a combination of C++, CUDA, python and Javascript. One of the major changes from the reference implementation of SCE is that we provide python bindings to the SCE method using pybind11 [37]. The C++/CUDA part of Mandrake which runs the entropic affinity preprocessing and modified SCE algorithm can be imported into any python program and called with 'triplet' sparse matrix data.

#### (i) Command line interface (python)

The full Mandrake executable is available as a python executable which includes genetic distance calculation, and plotting of the output. We include numerous progress meters for each stage of computation, as on large datasets estimating time or eliminating computationally impossible steps is a necessity. The package can be installed using conda, and we provide online documentation and examples at https://mandrake.readthedocs.io/en/latest/.

#### (ii) Optimization of GPU code (CUDA)

We optimized the CUDA code through multiple rounds of profiling, the results of which can be accessed with the datasets on Zenodo. Briefly, this resulted in the following changes:

— Use of a callback function to output the objective function at each iteration, so convergence can be monitored.
— Use of CUDA graphs to run each iteration, which eliminates overheads from calls to the CUDA API at every step.
— Reversing the strides of the embedding Y from row-major to column-major, which can sometimes coalesce memory accesses. Changing the strides back (to be compatible with numpy) is done in a new device kernel.
— Use of parallel reductions from the cub library to calculate the objective at the end of each step.
— Use of the wrapper classes from the dust package to manage device memory [31].
— Elimination of thread divergences within warps.
— Inclusion of 32-bit and 64-bit versions of the code (64-bit operations are slower and use more registers, and some devices can only emulate 64-bit floating point operations, which can decrease performance greatly).
— Storing each worker's random number generator state in registers, rather than writing to/from global memory whenever it is changed.
— Added compiler optimizations and loop unrolling.

#### (iii) Static web app (WebAssembly and Javascript)

We optimized a version of Mandrake for the web (https://gtonkinhill.github.io/mandrake-web). This is particularly important to improve accessibility for users who have less experience running and installing bioinformatics programs on the command line. We made use of the Emscripten compiler to convert a slightly modified version of the C++ code used in the python package to WebAssembly, which executes within the browser on the user's machine. This provides significant performance benefits over a pure Javascript-based implementation and allows the web application to achieve similar speeds as the command line version on small- to medium-sized datasets. As the support for multi-threading in WebAssembly is still experimental, the web application currently only supports runs on a single CPU, so the command line version is still recommended for very large datasets.

The static web application was created using the Hugo site generator and custom Javascript to interact with the compiled WebAssembly functions. A significant benefit of this approach is that once the website is loaded, there is no reliance on an internet connection and the entire analysis is run on the user's local machine. This ensures that the user's data is secure, as it is never uploaded, and which can be particularly useful in locations with poor internet connections where the uploading of any large dataset would be infeasible. It is also possible to run Mandrake-web entirely offline.

## 3. Results

### (a) Overview of Mandrake's design

**Figure 1.**
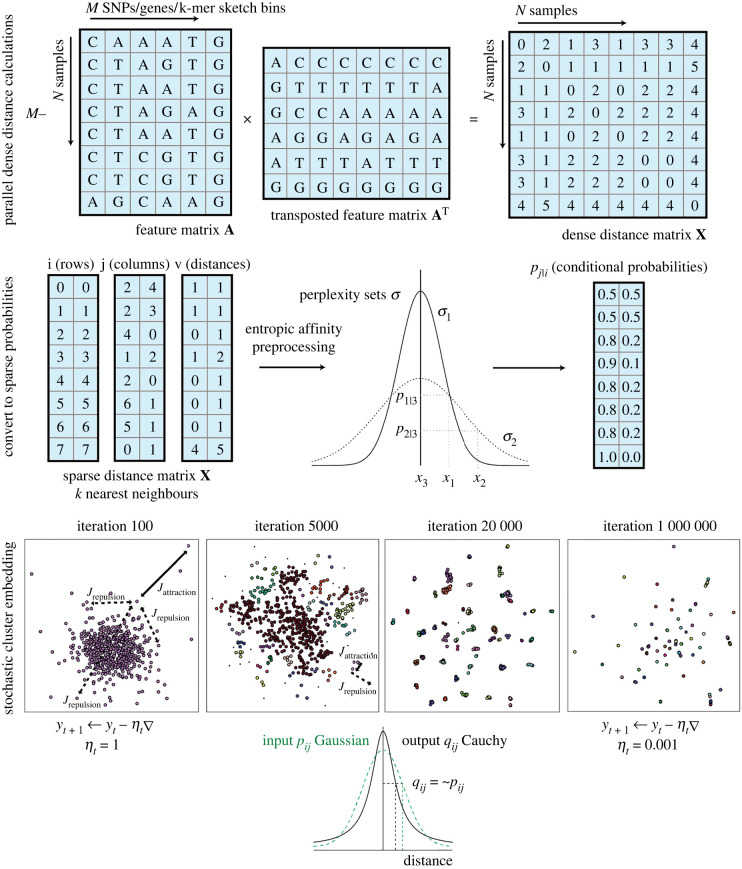
Overview of the Mandrake software. First, a genomic dataset, which may be a multiple sequence alignment, gene presence/absence or sequence sketches is used to calculate all pairwise distances between samples. Each entry in the distance matrix **X** is then the number of different features between each pair of samples. Each row of **X** is sorted, and the lowest *k* values (excluding self-matches of zero) are retained in triplet format. Entropic affinity converts these sparse distances to conditional probabilities, which can be thought of as the probability of selecting sample *x*_*j*_ as a neighbour, if probabilities are normally distributed. The user sets a perplexity parameter, which is used to set the variance of the distribution for each sample. SCE is run over a user-set number of iterations of SGD.

Figure 1 gives a graphical overview of the steps we use in Mandrake to create a low-dimensional embedding from genomic data. Pairwise genetic distances **X** between all samples are calculated from the genome data. Each element of **X**, of which there are *N*^2^, requires the comparison of *M* genomic features. This is typically the largest calculation in Mandrake, and we have highly optimized it and allow it to take advantage of many CPU cores where available. This makes calculation of distance matrices from up to millions of samples feasible. Each sample is reduced to the *k*-nearest neighbour distances on-the-fly to save space in memory. Note that although figure 1 removes identical distances for visual clarity, in our code we retain them. We then use entropic affinity, as described in the methods, to convert these distances into a conditional probability distribution, as described in the introduction. Figure 1 shows an example for sample *x*_3_, which has nearest neighbours *x*_1_ at one SNP away and *x*_2_ at two SNPs away. These are converted into probabilities using the height of Gaussian as shown, with a variance found to match the chosen perplexity through interval bisection.

SCE is then run; we make the user specify the number of iterations to run for and do not stop until this is reached. Some example frames across the SGD iterations are shown. At the start, points in the lower-dimensional space are randomly distributed, but are moved around more as the learning rate is higher. Later on, points are in clusters, and move smaller amounts along their gradient each step due to the lower learning rate. Some example attractive *J*_attraction_ and repulsive *J*_repulsion_ gradient steps are shown on the first two panels. Points are selected for attraction more frequently if they have a higher conditional probability. This has the effect that within a cluster (close in the higher dimension; higher conditional probabilities), points are pushed together. Repulsion is between any pair of points, which at later stages of the algorithm repulses clusters from one another, with the attractive force keeping the cluster together.

Applying SGD to *D* tries to make the input distribution (the conditional probabilities *p_j_*_|*i*_) as similar to possible to the output distribution (set by a Cauchy distribution *q_ij_*). This is shown at the bottom of figure 1—an example with two points with the same input and output probability on the *y*-axis is a small distance apart on the *x*-axis. Therefore, close distances in the higher-dimensional space of **X** will also be close in the lower-dimensional space **Y**. The heavy tails of the output Cauchy (black, solid distribution) apply less penalization if smaller input probabilities are further apart than the tails of a Gaussian distribution (green, dashed distribution).

Our resulting implementation runs in a few hours, on up to around one million samples (table 1). Some variation in runtimes not directly proportional to the number of iterations is observed, this is typically due to setting a number of workers to aim for a maximum 10% clash rate on each dataset, such that efficiency increased in the larger datasets. Our web app is responsive up to the range of 10 000 samples, which completes in around a minute, depending on the input data type.

**Table 1.** Resource usage for Mandrake on datasets used. The first row shows use of the static web app on a single core. All other reported times used 60 CPU cores, and where applicable an Nvidia 3090 RTX GPU was with double precision (fp64) or single precision (fp32). Note, GPU distance calculations are supported for sketches, but the table reports the CPU time only. The HIV pol gene alignment used was a random 5000 subset from the Los Alamos public database [38].

dataset	no. samples	distance calculation	maximum memory	iterations used	SCE time (CPU)	SCE time (GPU)
HIV-1 *pol* gene alignment	5000	35 s (web)	NA	1 × 10^6^	11 s (web)	n.a.
*S. pneumoniae* accessory genome	20 047	3 min	6.5 Gb (host)/0.35 Gb (GPU)	3 × 10^8^	7.8 min	5 s (fp64)
SRA bacterial assemblies	661 406	3 h	7 Gb (host)/3 Gb (GPU)	5 × 10^11^	186 h	1.3 h (fp64)
SARS-CoV-2 alignment	941 981	101 h	26 Gb (host)/13 Gb (GPU)	1 × 10^12^	372 h	2.8 h (fp64)/2.3 h (fp32)

Before interpreting our results on different datasets, we recap some key features of nonlinear embeddings [39]:

— Cluster sizes in the embedding space do not relate to the number of points in the cluster, or its genetic diversity. In SCE particularly, many points will be heavily overplotted, and the density plot should be used for determining the number of samples in one region.
— Distances between clusters do not correspond to their genetic distances. Two well-separated clusters, close together, are not necessarily more genetically similar than two well-separated clusters at opposite ends of the plot.
— Perplexity can greatly affect results, and runs at a few different perplexities should typically be attempted. At lower perplexities, structure can sometimes be found where there is none. Complex topological relationships are generally not expected in genetic data, but where these may exist (in the presence of extensive horizontal gene transfer) multiple perplexity runs may be able to find these.

### (b) Mandrake embeddings accurately reflect simulated population structure

**Figure 2.**
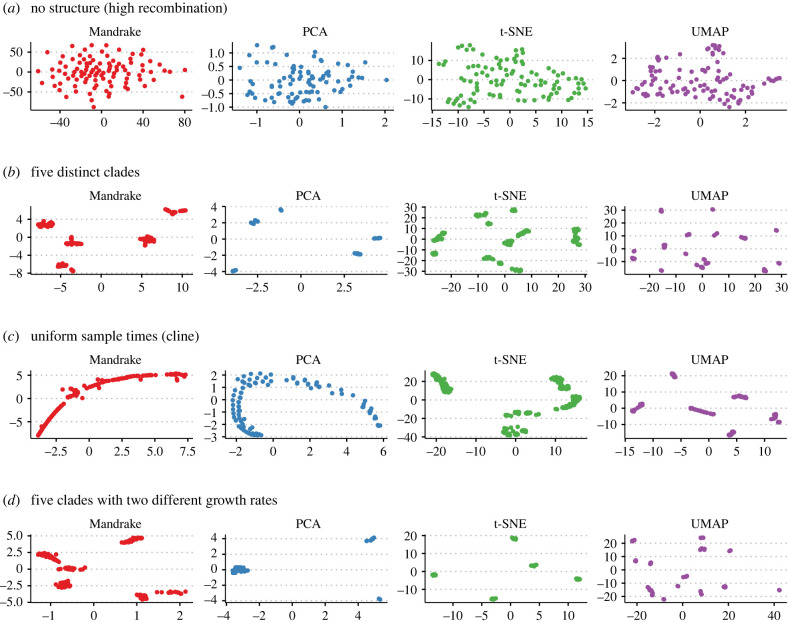
Two-dimensional embeddings from Mandrake, PCA, t-SNE and UMAP on simulated genomes. (*a*) Simulations with a high recombination rate and consequently no shared structure. (*b*) Five metapopulations, representing strains/clades. (*c*) A single population with a constant mutation rate, sampled uniformly over time. (*d*) As (*b*), but with the metapopulations split into two groups, between which their growth rates vary by 10^3^.

To verify that Mandrake was able to produce an embedding that accurately described the underlying population structure of a dataset, we simulated five distinct scenarios using a coalescent model with recombination, as implemented in the R programs Coala and scrm [40,41]. We then generated two-dimensional embeddings of the resulting SNP matrices using Mandrake, PCA, t-SNE and UMAP (figure 2). The first simulation (figure 2*a*) included a very high recombination rate, which acted to remove any underlying structure in the dataset: all four embedding algorithms correctly produced visualizations with no obvious structure or clusters. Mandrake and PCA produced the most accurate embeddings on the following two simulations. The first consisted of five distinct clades and the second a single population sampled uniformly over time which produced genetic distances along a gradient or cline (figure 2*b,c*). To demonstrate the utility of nonlinear embeddings, we also compared each method on a further dataset involving five clades, but with each clade having one of two significantly different growth rates (figure 2*d*). In this case, PCA was able to separate the two clades with larger growth rates, but did not distinguish the three distinct clades with smaller growth rates which appear clumped together in the left-hand side of the plot. Conversely, both Mandrake and t-SNE were able to distinguish all five clades. Although the Mandrake embedding slightly over partitioned one clade in this case, it generally outperformed the other three methods when considering all four scenarios. In particular, we found that the UMAP embedding rarely reflected the underlying simulated population structure.

### (c) Embedding the accessory genome of 20 k *Streptococcus pneumoniae*

To demonstrate the ability of the SCE embedding to identify meaningful clusters within a large dataset, we first considered a collection of 20 047 *S. pneumoniae* genomes that consisted of a subset of high-quality genomes from the Global Pneumococcal Sequencing project and two other pneumococcal genome surveillance studies [4,42–44]. *S. pneumoniae* is a highly recombinant bacterial species with an extensive accessory genome that has been shown to be highly structured [45,46]. This makes it a good example for investigating the ability of Mandrake to identify clusters from a gene presence/absence matrix.

We first inferred a pangenome gene presence/absence matrix using Panaroo v.1.2 [47]. This resulted in a binary matrix consisting of 27 322 features (genes) and 20 047 genomes, which were used as input to Mandrake.

**Figure 3.**
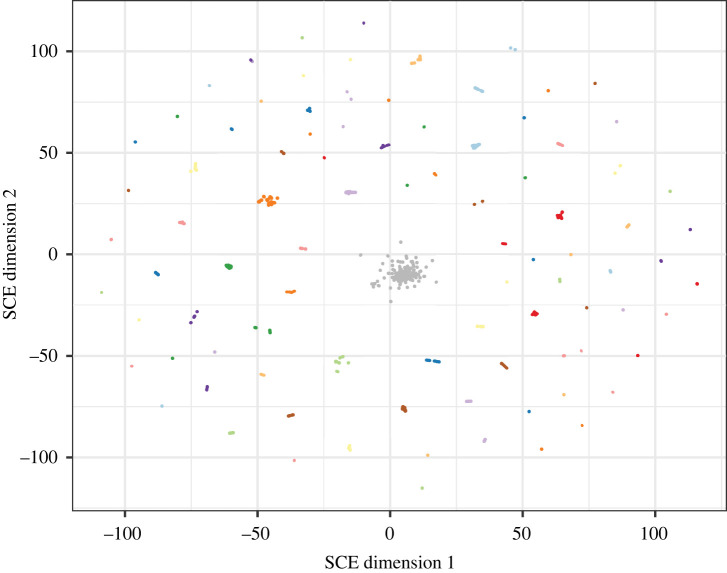
A two-dimensional embedding calculated using Mandrake from the accessory gene presence/absence matrix of 20 047 pneumococcal genomes. Points are coloured by the underlying GPSC to which they belong. Those GPSCs with fewer than 50 isolates are coloured in grey.

Figure 3 and electronic supplementary material, video S1 indicate the resulting embedding, with points coloured according to which of the Global Pneumococcal Sequencing Clusters (GPSCs) each genome belonged to [4,42]. These clusters are defined by PopPUNK and use both core and accessory distances, and although these distances are strongly correlated in encapsulated *S. pneumoniae,* they are not necessarily a direct comparison or ground truth for a purely accessory clustering of this dataset. However, they are commonly used for epidemiological and evolutionary analysis, so are an instructive comparison nevertheless. Those clusters with fewer than 50 genomes are coloured in grey, with the Mandrake embedding placing them together in a single large group. This is similar to the behaviour of other clustering algorithms such as BAPS where outlying genomes are often grouped together into a single cluster representing the broader genomic background [38,48]. To compare the observed clustering in the 2D embedding with the underlying GPSCs, we calculated both the rand index and the adjusted mutual information (AMI) after first clustering the embedded points using HDBSCAN [36]. Both the Rand index and the AMI are measures of similarity between two clustering assignments and provide an indication of the accuracy with which one clustering predicts the other. In general, the AMI provides more accurate estimates when the underlying cluster sizes differ significantly. The Mandrake embedding was found to have an Rand index of 0.987 and an AMI of 0.085, which was similar but still higher than that found using common alternative embeddings including UMAP, t-SNE and PCA (electronic supplementary material, table S1). This suggests that Mandrake is able to produce a biologically meaningful embedding quickly using the presence and absence of accessory genes as input.

### (d) Embedding 650 k bacterial genome assemblies from public databases

**Figure 4.**
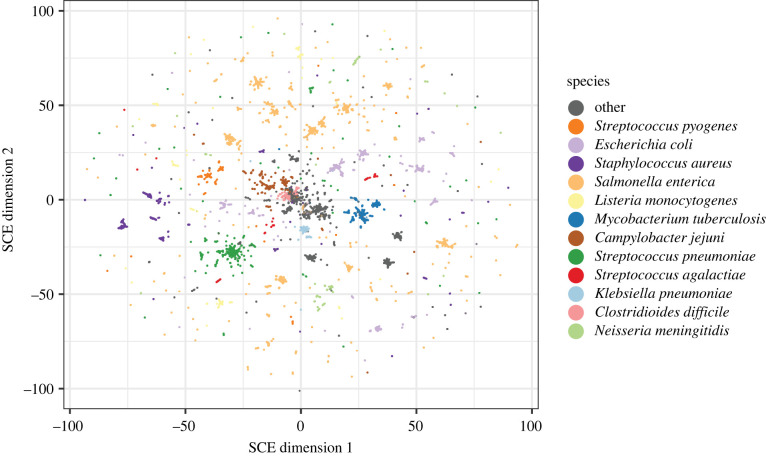
A Mandrake embedding of 662 406 bacterial genomes from the SRA with Jaccard distances calculated using sketches of 14-mers. Species with at least 10 000 genomes in the dataset are coloured.

We then used SCE to search for structure across the space of highly diverse bacterial genomes. A recent analysis produced curated and assembled bacterial samples from the SRA database, producing 661406 high-quality bacterial genomes [49]. We downloaded these assemblies from http://ftp.ebi.ac.uk/pub/databases/ENA2018-bacteria-661k/ and sketched their 14-mers. We then used these sketches to calculate Jaccard distances between 14-mers, which we used to produce a sparse matrix with the 100 nearest neighbours. Using this, we ran Mandrake for 5 × 10^11^ iterations using 65 536 workers on a GPU, which took 2 h. The objective stabilized around halfway through the run, and the resulting embedding can be seen in figure 4 and an animation of the SCE iterations in electronic supplementary material, video S2.

We found that the most common species formed clear clusters in the embedding space, with the exception of *Closteroides difficile* in the centre of the space, which overlaps with many other gut pathogens. We hypothesized that this may be due to gut samples where the assembled sequence contained multiple species, but just the most abundant species were reported. We analysed the overall species diversity and proportion of *C. difficile* sequence reported for the samples in this dataset and plotted this section of the embedding (electronic supplementary material, figure S1). This shows that the central cluster is composed mostly of mixed samples, which would be expected to be difficult to cluster, and that most of the *C. difficile*, when viewed at higher resolution, are separated from these other samples. Looking at panels B and C, one can see that a number of the *C. difficile* samples are mixed with other species, which is probably why they have been placed in this large cluster in the plot, pulling other similar clusters with them.

Most species were split into multiple clusters, possibly representing strains within species, or subspecies. We investigated a few notable examples further. *Salmonella enterica* consists of multiple subspecies and multiple strains that can be clustered with the HierCC scheme using HC2850 and HC900, respectively [12,50]. We coloured the embedding by these classifications (electronic supplementary material, figure S2), using samples assigned to these schemes in Enterobase [7]. 98.5% are HC2850 2 (subspecies I), which are split over multiple clusters; the remaining HC types are in separate clusters on the plot. HC900 is more similar to the clusters found in the plot, with some loss of information in smaller clusters (similar to the GPSC analysis above) and some larger clusters further split up. The clusters therefore correspond to a resolution somewhat finer than HC900. Very similar findings are found running on the *Salmonella* genomes alone, showing that Mandrake finds clusters corresponding to biological entities within species, both when run across and between species.

Some other interesting examples include *Listeria monocytogenes*, which has genetically distinct major lineages [51] and appears as separate clusters spread around the embedding that map onto these, but with further clusters within each lineage (electronic supplementary material, figure S3). *Mycobacterium tuberculosis* is split into 5–10 clusters, which broadly correspond to its major lineages defined by *TBProfiler* [52], though with some differences (electronic supplementary material, figure S4). The nonlinear nature of the embedding is therefore able to simultaneously capture structure across a range of genetic scales: strains, subspecies and species.

We also note a useful example for interpreting the plot here: although *M. tuberculosis* has a larger radius in the embedding than *Streptococcus agalactiae*, the former harbours much less genetic diversity overall. This is an example of the first point in our caveats on reading embeddings: cluster sizes in the embedding space do not relate to the number of points in the cluster, or its genetic diversity. However, the *M. tuberculosis* clusters are close together in the space, so between cluster distances sometimes retain meaning.

### (e) Embedding 1M SARS-CoV-2 genome alignments from public databases

We next considered Mandrake's ability to embed highly similar genomes into clusters by running the algorithm in its multiple sequence alignment mode on a cleaned subset of 941 981 SARS-CoV-2 genomes downloaded from the European Nucleotide Archive (ENA; https://www.covid19dataportal.org). Of the original 977 048 genomes downloaded, we filtered out 35 067 that had a length less than 90% of the Wuhan.1 reference genome or were made up of more than 5% ambiguous nucleotide calls. Each genome was assigned to a SARS-CoV-2 lineage using pangolin [5,53]. After generating a multiple sequence alignment of the genomes using MAFFT v.7.487 [54], we ran Mandrake in its ‘alignment’ mode, which calculates the pairwise Hamming distance between genomes ignoring ambiguous base calls. Mandrake was run for 1 × 10^12^ iterations on a GPU with 94 976 workers, which took 3.7 h.

**Figure 5.**
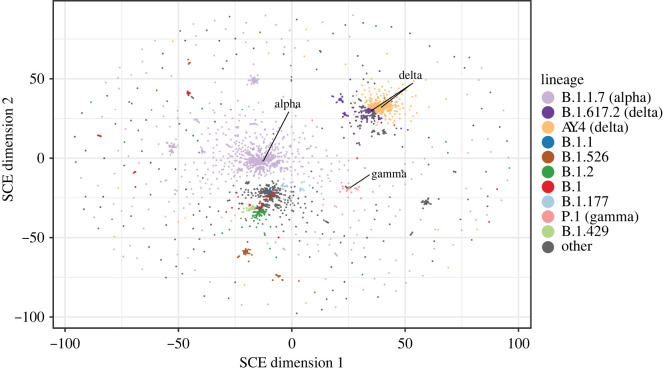
A Mandrake embedding of 941 981 SARS-CoV-2 genomes downloaded from the ENA. Points corresponding to genomes within large Pangolin lineages (more than 10 000 members) are coloured and variants of concern are manually labelled.

The resulting embedding is shown in figure 5 and electronic supplementary material, video S3, with the major SARS-CoV-2 lineages comprising more than 10 000 genomes assigned different colours. Interestingly, the major variants of concern including the Delta and Alpha lineages are clearly visible in the embedding, indicating that Mandrake is able to identify biologically meaningful structure within very large set of highly similar genomes. The delta variable of concern (VOC) includes two major Pangolin lineages that have been placed together in the Mandrake embedding. We note the presence of a number of outlying points that have been assigned to lineages. This may either reflect additional sub-structure within the lineages or potential sequencing artefacts, or be a limitation of the method.

## 4. Discussion

The population genomics of pathogens is currently experiencing an unprecedented pace of genome sequencing on a global scale, which poses a challenge to many standard workflows for data analysis. As many downstream epidemiological or evolutionary analyses work within identified clusters, the first tasks in population genomics workflows are to understand the population structure and the extent of clustering of the input genomes, both of which are difficult to do with increasing data size. While some new highly scalable methods have been developed for this task, they are frequently species-specific [55]. Doing this in a manner that can be visualized is particularly helpful, especially given the high dimensions and complex relationships inherent in genomic datasets.

In this article, we have presented the Mandrake software, which was designed to meet these particular needs and offers programmatic plotting options and interactive exploration of the data. Our current software architecture scales well to even the largest available contemporary pathogen genomics datasets. However, in future, it would clearly benefit from reducing the quadratic computational complexity of the input genome distance calculations, which could for example be achieved with subsampling of the data by picking representative samples among highly similar genomes. This has been achieved in other packages by assuming that genetic distances generally obey the triangle inequality [14,56].

We have shown that Mandrake is able to find genetic structure across a range of diversities: via simulations of distinct population structures within SARS-CoV-2 genomes, which vary fairly continuously with a low density of SNPs, through the gene content variation within the global *S. pneumoniae* population, to the current corpus of sequenced bacteria. In all of these examples, no labelling of the input was needed and clusters that correspond to useful biological units were automatically found. In some species in the bacterial collections, Mandrake was even able to simultaneously find clusters within a species, while still clustering different species correctly. Multiple orders of magnitude of genetic diversity can therefore be exposed in the same plot due to the local nature of the nearest-neighbour approach. However, clusters formed by these plots are generally not expected to be competitive with species-specific schemes, which have usually been curated and optimized to find useful clusters, and that also have more favourable properties in terms of assigning new samples to existing clusters and maintaining consistent nomenclature [12,14].

Another interesting opportunity for further research and development stems from the challenge of optimizing output plots using models of human perception. Here, we used user-guided training in SCE to determine a parameter *s*, which governs the display of clusters in the output embedding. Recent results in perception modelling for visualization have demonstrated notable improvements over default software options for scatterplots, where optimized designs can much better adapt to an increase in data density [57]. There are multiple display parameters that could be adjusted in order to give a human expert an enhanced view into the data structure, such as the marker size, their opacity and colours. Optimization of such parameters using models of human cognition has the potential to resolve the visualization challenges arising from extremely high dimensionality of the data, not only for cluster embedding as considered here, but also for other complex objects such as phylogenetic trees. Several successful examples of using approximate Bayesian computation in cognitive model inference [58,59] suggest that this approach could be fruitful for making improved displays of high-dimensional population genomic data. Further improvements could also still be made to the actual implementation of our output, by including common techniques such as automatically grouping data when zooming, and selecting data from a table—though these are a challenge at this scale of data.

Nevertheless, we have been able to make Mandrake useful across a range of scales and to a range of users, scaling from within-browser analysis, through multicore CPU use on the command line, up to high-power graphics cards. The functions provided may also serve as a basis for the analysis of pairwise relationships between genomic data in other tools, such as phylogenetics [60], selection analysis [61] and mathematical modelling [62].

## Data Availability

Code: https://github.com/johnlees/mandrake and https://github.com/gtonkinhill/mandrake-web. Documentation: https://mandrake.readthedocs.io/en/latest/. Archived code: https://dx.doi.org/10.5281/zenodo.5579270. Commands used for analysis: https://github.com/gtonkinhill/mandrake_manuscript. Datasets: https://dx.doi.org/10.5281/zenodo.5572316.

## References

[1] Armstrong GL, MacCannell DR, Taylor J, Carleton HA, Neuhaus EB, Bradbury RS, Posey JE, Gwinn M. 2019 Pathogen genomics in public health. N. Engl. J. Med. **381**, 2569-2580. (10.1056/NEJMsr1813907) 31881145 PMC7008580

[2] Brown B, Allard M, Bazaco MC, Blankenship J, Minor T. 2021 An economic evaluation of the whole genome sequencing source tracking program in the U.S. PLoS ONE **16**, e0258262. (10.1371/journal.pone.0258262) 34614029 PMC8494326

[3] Oude MBB, Worp N, Nieuwenhuijse DF, Sikkema RS, Haagmans B, Fouchier RAM, Koopmans M. 2021 The next phase of SARS-CoV-2 surveillance: real-time molecular epidemiology. Nat. Med. **27**, 1518-1524. (10.1038/s41591-021-01472-w) 34504335

[4] Gladstone RA et al. 2019 International genomic definition of pneumococcal lineages, to contextualise disease, antibiotic resistance and vaccine impact. EBioMedicine **43**, 338-346. (10.1016/j.ebiom.2019.04.021) 31003929 PMC6557916

[5] O'Toole Á et al. 2021 Assignment of epidemiological lineages in an emerging pandemic using the pangolin tool. Virus Evol. **7**, veab064. (10.1093/ve/veab064) 34527285 PMC8344591

[6] Croucher NJ, Didelot X. 2015 The application of genomics to tracing bacterial pathogen transmission. Curr. Opin. Microbiol. **23**, 62-67. (10.1016/j.mib.2014.11.004) 25461574

[7] Zhou Z, Alikhan NF, Mohamed K, Fan Y, Achtman M. 2020 The EnteroBase user's guide, with case studies on *Salmonella* transmissions, *Yersinia pestis* phylogeny, and *Escherichia* core genomic diversity. Genome Res. **30**, 138-152. (10.1101/gr.251678.119) 31809257 PMC6961584

[8] van Belkum A et al. 2007 Guidelines for the validation and application of typing methods for use in bacterial epidemiology. Clin. Microbiol. Infect. **13**(Suppl. 3), 1-46. (10.1111/j.1469-0691.2007.01786.x) 17716294

[9] Tonkin-Hill G, Lees JA, Bentley SD, Frost SDW, Corander J. 2018 RhierBAPS: an R implementation of the population clustering algorithm hierBAPS. Wellcome Open Res. **3**, 93. (10.12688/wellcomeopenres.14694.1) 30345380 PMC6178908

[10] Achtman M et al. 2012 Multilocus sequence typing as a replacement for serotyping in *Salmonella enterica*. PLoS Pathog. **8**, e1002776. (10.1371/journal.ppat.1002776) 22737074 PMC3380943

[11] Feil EJ, Enright MC. 2004 Analyses of clonality and the evolution of bacterial pathogens. Curr. Opin. Microbiol. **7**, 308-313. (10.1016/j.mib.2004.04.002) 15196500

[12] Zhou Z, Charlesworth J, Achtman M. 2021 HierCC: a multi-level clustering scheme for population assignments based on core genome MLST. Bioinformatics **37**, 3645-3646. (10.1093/bioinformatics/btab234) PMC8545296 33823553

[13] Jombart T, Devillard S, Balloux F. 2010 Discriminant analysis of principal components: a new method for the analysis of genetically structured populations. BMC Genet. **11**, 94. (10.1186/1471-2156-11-94) 20950446 PMC2973851

[14] Lees JA et al. 2019 Fast and flexible bacterial genomic epidemiology with PopPUNK. Genome Res. **29**, 304-316. (10.1101/gr.241455.118) 30679308 PMC6360808

[15] Black A, MacCannell DR, Sibley TR, Bedford T. 2020 Ten recommendations for supporting open pathogen genomic analysis in public health. Nat. Med. **26**, 832-841. (10.1038/s41591-020-0935-z) 32528156 PMC7363500

[16] Maaten L, Hinton G. 2008 Visualizing data using t-SNE. J. Mach. Learn. Res. **9**, 2579-2605.

[17] van der Maaten L. 2014 Accelerating t-SNE using tree-based algorithms. J. Mach. Learn. Res. **15**, 3221-3245. (10.5555/2627435.2697068)

[18] Abudahab K, Prada JM, Yang Z, Bentley SD, Croucher NJ, Corander J, Aanensen DM. 2018 PANINI: pangenome neighbour identification for bacterial populations. Microb. Genom. **4**, 220. (10.1099/mgen.0.000220) PMC6521588 30465642

[19] Becht E, McInnes L, Healy J, Dutertre CA, Kwok IWH, Ng LG, Ginhoux F, Newell EW. 2018 Dimensionality reduction for visualizing single-cell data using UMAP. Nat. Biotechnol. **37**, 38-44. (10.1038/nbt.4314) 30531897

[20] Kobak D, Berens P. 2019 The art of using t-SNE for single-cell transcriptomics. Nat. Commun. **10**, 1-14. (10.1038/s41467-019-13056-x) 31780648 PMC6882829

[21] Diaz-Papkovich A, Anderson-Trocmé L, Gravel S. 2021 A review of UMAP in population genetics. J. Hum. Genet. **66**, 85-91. (10.1038/s10038-020-00851-4) 33057159 PMC7728596

[22] Yang Z, Chen Y, Sedov D, Kaski S, Corander J. 2021 Stochastic cluster embedding. arXiv [cs.LG]. See http://arxiv.org/abs/2108.08003

[23] Lees JA, Mai TT, Galardini M, Wheeler NE, Horsfield ST, Parkhill J, Corander J. 2020 Improved prediction of bacterial genotype-phenotype associations using interpretable pangenome-spanning regressions. MBio **11**. (10.1128/mBio.01344-20) PMC7343994 32636251

[24] Ondov BD, Treangen TJ, Melsted P, Mallonee AB, Bergman NH, Koren S, Phillippy AM. 2016 Mash: fast genome and metagenome distance estimation using MinHash. Genome Biol. **17**, 1-14. (10.1186/s13059-016-0997-x) 27323842 PMC4915045

[25] Zhao X. 2019 BinDash, software for fast genome distance estimation on a typical personal laptop. Bioinformatics **35**, 671-673. (10.1093/bioinformatics/bty651) 30052763

[26] Vladymyrov M, Carreira-Perpinan M. 2013 Entropic affinities: properties and efficient numerical computation. In Proc. of the 30th Int. Conf. on Machine Learning (eds S Dasgupta, D McAllester), pp. 477-485. Atlanta, GA: PMLR.

[27] Pedregosa F et al. 2011 Scikit-learn: machine learning in Python. J. Mach. Learn. Res. **12**, 2825-2830.

[28] Bottou L et al. 1991 Stochastic gradient learning in neural networks. Proc. Neuro-Nımes **91**, 12.

[29] Walker AJ. 1977 An efficient method for generating discrete random variables with general distributions. ACM Trans. Math. Softw. **3**, 253-256. (10.1145/355744.355749)

[30] Knuth DE. 1997 The art of computer programming, volume 2 (3rd ed.): seminumerical algorithms. Boston, MA: Addison-Wesley Longman Publishing Co., Inc.

[31] FitzJohn RG et al. 2021 Reproducible parallel inference and simulation of stochastic state space models using odin, dust, and mcstate. Wellcome Open Res. **5**, 288. (10.12688/wellcomeopenres.16466.2) 34761122 PMC8552050

[32] Blackman D, Vigna S. 2018 Scrambled linear pseudorandom number generators. arXiv [cs.DS]. See http://arxiv.org/abs/1805.01407.

[33] Inc. PT. 2016 Collaborative data science. Montreal, QC: Plotly Technologies Inc. See https://plot.ly

[34] Hunter JD. 2007 Matplotlib: a 2D graphics environment. Comput. Sci. Eng. **9**, 90-95. (10.1109/MCSE.2007.55)

[35] Argimón S et al. 2016 Microreact: visualizing and sharing data for genomic epidemiology and phylogeography. Microb. Genom. **2**, e000093. (10.1099/mgen.0.000093) 28348833 PMC5320705

[36] McInnes L, Healy J, Astels S. 2017 hdbscan: hierarchical density based clustering. J. Open Source Software **2**, 205. (10.21105/joss.00205)

[37] Jakob W, Rhinelander J, Moldovan D. 2017 pybind11 -- Seamless operability between C++11 and Python. (See https://github.com/pybind/pybind11.)

[38] Tonkin-Hill G, Lees JA, Bentley SD, Frost SDW, Corander J. 2019 Fast hierarchical Bayesian analysis of population structure. Nucleic Acids Res. **47**, 5539-5549. (10.1093/nar/gkz361) 31076776 PMC6582336

[39] Wattenberg M, Viégas F, Johnson I. 2016 How to use t-SNE effectively. Distill **1**, 2. (10.23915/distill.00002)

[40] Staab PR, Metzler D. 2016 Coala: an R framework for coalescent simulation. Bioinformatics **32**, 1903-1904. (10.1093/bioinformatics/btw098) 27153679

[41] Staab PR, Zhu S, Metzler D, Lunter G. 2015 scrm: efficiently simulating long sequences using the approximated coalescent with recombination. Bioinformatics **31**, 1680-1682. (10.1093/bioinformatics/btu861) 25596205 PMC4426833

[42] Lo SW et al. 2019 Pneumococcal lineages associated with serotype replacement and antibiotic resistance in childhood invasive pneumococcal disease in the post-PCV13 era: an international whole-genome sequencing study. Lancet Infect. Dis. **19**, 759-769. (10.1016/S1473-3099(19)30297-X) 31196809 PMC7641901

[43] Chewapreecha C et al. 2014 Dense genomic sampling identifies highways of pneumococcal recombination. Nat. Genet. **46**, 305-309. (10.1038/ng.2895) 24509479 PMC3970364

[44] Croucher NJ et al. 2013 Population genomics of post-vaccine changes in pneumococcal epidemiology. Nat. Genet. **45**, 656-663. (10.1038/ng.2625) 23644493 PMC3725542

[45] Corander J et al. 2017 Frequency-dependent selection in vaccine-associated pneumococcal population dynamics. Nat. Ecol. Evol. **1**, 1950-1960. (10.1038/s41559-017-0337-x) 29038424 PMC5708525

[46] Azarian T et al. 2020 Frequency-dependent selection can forecast evolution in *Streptococcus pneumoniae*. PLoS Biol. **18**, e3000878. (10.1371/journal.pbio.3000878) 33091022 PMC7580979

[47] Tonkin-Hill G et al. 2020 Producing polished prokaryotic pangenomes with the Panaroo pipeline. Genome Biol. **21**, 180. (10.1186/s13059-020-02090-4) 32698896 PMC7376924

[48] Cheng L, Connor TR, Sirén J, Aanensen DM, Corander J. 2013 Hierarchical and spatially explicit clustering of DNA sequences with BAPS software. Mol. Biol. Evol. **30**, 1224-1228. (10.1093/molbev/mst028) 23408797 PMC3670731

[49] Blackwell GA, Hunt M, Malone KM, Lima L, Horesh G, Alako BTF, Thomson NR, Iqbal Z. 2021 Exploring bacterial diversity via a curated and searchable snapshot of archived DNA sequences. PLoS Biol. **19**, e3001421. (10.1371/journal.pbio.3001421) 34752446 PMC8577725

[50] Alikhan NF, Zhou Z, Sergeant MJ, Achtman M. 2018 A genomic overview of the population structure of *Salmonella*. PLoS Genet. **14**, e1007261. (10.1371/journal.pgen.1007261) 29621240 PMC5886390

[51] Moura A et al. 2016 Whole genome-based population biology and epidemiological surveillance of *Listeria monocytogenes*. Nat. Microbiol. **2**, 16185. (10.1038/nmicrobiol.2016.185) 27723724 PMC8903085

[52] Phelan JE et al. 2019 Integrating informatics tools and portable sequencing technology for rapid detection of resistance to anti-tuberculous drugs. Genome Med. **11**, 41. (10.1186/s13073-019-0650-x) 31234910 PMC6591855

[53] Rambaut A, Holmes EC, O'Toole Á, Hill V, McCrone JT, Ruis C, du Plessis L, Pybus OG. 2020 A dynamic nomenclature proposal for SARS-CoV-2 lineages to assist genomic epidemiology. Nat. Microbiol. **5**, 1403-1407. (10.1038/s41564-020-0770-5) 32669681 PMC7610519

[54] Katoh K, Standley DM. 2013 MAFFT multiple sequence alignment software version 7: improvements in performance and usability. Mol. Biol. Evol. **30**, 772-780. 23329690 10.1093/molbev/mst010 PMC3603318

[55] Turakhia Y et al. 2021 Ultrafast sample placement on Existing tRees (UShER) enables real-time phylogenetics for the SARS-CoV-2 pandemic. Nat. Genet. **53**, 809-816. (10.1038/s41588-021-00862-7) 33972780 PMC9248294

[56] Steinegger M, Söding J. 2018 Clustering huge protein sequence sets in linear time. Nat. Commun. **9**, 2542. (10.1038/s41467-018-04964-5) 29959318 PMC6026198

[57] Micallef L, Palmas G, Oulasvirta A, Weinkauf T. 2017 Towards perceptual optimization of the visual design of scatterplots. IEEE Trans. Vis. Comput. Graph **23**, 1588-1599. (10.1109/TVCG.2017.2674978) 28252407

[58] Gebhardt C, Oulasvirta A, Hilliges O. 2021 Hierarchical reinforcement learning explains task interleaving behavior. Comput. Brain Behav. **4**, 284-304. (10.1007/s42113-020-00093-9)

[59] Kangasrääsiö A, Jokinen JPP, Oulasvirta A, Howes A, Kaski S. 2019 Parameter inference for computational cognitive models with approximate Bayesian computation. Cogn. Sci. **43**, e12738. (10.1111/cogs.12738) 31204797 PMC6593436

[60] Nguyen LT, Schmidt HA, von Haeseler A, Minh BQ. 2015 IQ-TREE: a fast and effective stochastic algorithm for estimating maximum-likelihood phylogenies. Mol. Biol. Evol. **32**, 268-274. (10.1093/molbev/msu300) 25371430 PMC4271533

[61] Frost SDW, Magalis BR, Kosakovsky Pond SL. 2018 Neutral theory and rapidly evolving viral pathogens. Mol. Biol. Evol. **35**, 1348-1354. (10.1093/molbev/msy088) 29688481 PMC6279309

[62] Vöhringer HS et al. 2021 Genomic reconstruction of the SARS-CoV-2 epidemic in England. Nature **600**, 506-511. (10.1038/s41586-021-04069-y) 34649268 PMC8674138

